# An unusual presentation of chordoma as a pyloric ring submucosal tumor: The first case report of a primary gastrointestinal lesion in humans

**DOI:** 10.1016/j.ijscr.2022.107032

**Published:** 2022-04-06

**Authors:** Ryotaro Hashizume, Shinsuke Matsuda, Moritaka Nagai, Kazuki Hirata, Hiroshi Imai, Ryoji Kushima

**Affiliations:** a Department of Pathology and Matrix Biology, Mie University Graduate School of Medicine, Tsu, Mie 514-8507, Japan; b Department of Genomic Medicine, Mie University Hospital, Tsu, Mie 514-8507, Japan; c Department of Surgery, Nagai Hospital, Tsu, Mie 514-8508, Japan; d Department of Pathology Laboratory, Nagai Hospital, Tsu, Mie 514-8508, Japan; e Pathology Division, Mie University Hospital, Tsu, Mie 514-8508, Japan; f Department of Pathology, Undergraduate School of Medicine, Shiga University of Medical Science, Otsu, Shiga 520-2192, Japan; g Pathology Division, National Cancer Center Hospital, Tokyo 104-0045, Japan

**Keywords:** Chordoma, Gastrointestinal, Sub-mucosal tumor

## Abstract

**Introduction and importance:**

Chordomas are rare malignant bone neoplasms that are presumed to arise from chordal remnants in the fetal stage and typically occur along the axial skeleton. The extra-skeletal chordomas reported to date include soft tissue of the extremities and nasopharynx. Chordoma arising from the gastrointestinal wall has not been previously described.

**Case presentation:**

We report on a 42-year-old man with primary chordoma presenting as a gastroduodenal submucosal tumor centered on the pyloric ring. The patient was consistently asymptomatic, and the tumor was an incidental finding. However, during a follow-up at approximately 1.6 years, an increase in tumor size was identified on computed tomography (CT), and surgical resection was performed without a definite pathologic diagnosis. The patient was successfully treated with distal gastrectomy, and the histological diagnosis was a conventional chordoma. The diagnosis was confirmed via immunohistochemical staining for brachyury, pan-cytokeratin, S-100, and SOX9. Postoperative CT and magnetic resonance imaging revealed no recurrence or metastasis during the 1.5-year follow-up period.

**Clinical discussion:**

Primary chordomas of the digestive tract are rare. Embryologic development of the notochord does not explain the existence of remnants in the gastrointestinal wall. Moreover, notochordal remnants, as precursors of chordoma, were not identified in the current case. The gastroduodenal chordoma may not have originated from embryonic notochordal remnants but through aberrant brachyury activation without a notochordal precursor.

**Conclusion:**

We report the first case of primary gastrointestinal chordoma in humans. The tumor was completely removed surgically, without postoperative recurrence.

## Highlights

• This is a case of primary gastrointestinal submucosal chordoma in a 42-year-old man
• Chordoma is a rare bony tumor that most frequently affects the axial skeleton
• Extra-skeletal chordomas are extremely rare in any species
• This is the first report of primary chordoma of the digestive tract in humans

## 1 Introduction

Chordomas are rare malignant tumors that presumably originate from the remnants of the primitive notochord with a high propensity for local recurrence. These usually arise from the axial skeleton of the sacrum, skull base, or mobile spine [1]. Less frequently, chordomas may also arise from extra-axial locations. Several extra-skeletal chordomas have been reported, although they are exceptionally rare. However, to date, no primary chordoma of the gastrointestinal wall has been reported. The most common primary gastrointestinal submucosal tumors include gastrointestinal stromal tumors (GIST), leiomyomas, lipomas, carcinoids, granular-cell tumors, glomus tumors, schwannomas, Brunner's gland hamartomas, and pancreatic rests [2]. Except for metastatic lesions, chordomas are not usually considered gastrointestinal submucosal tumors. In line with the Consensus-based Surgical Case Reporting Guideline (SCARE) criteria [3], we present a case of a 42-year-old man with a primary chordoma located at the gastroduodenal submucosa.

## 2 Presentation of case

**Fig. 1:**
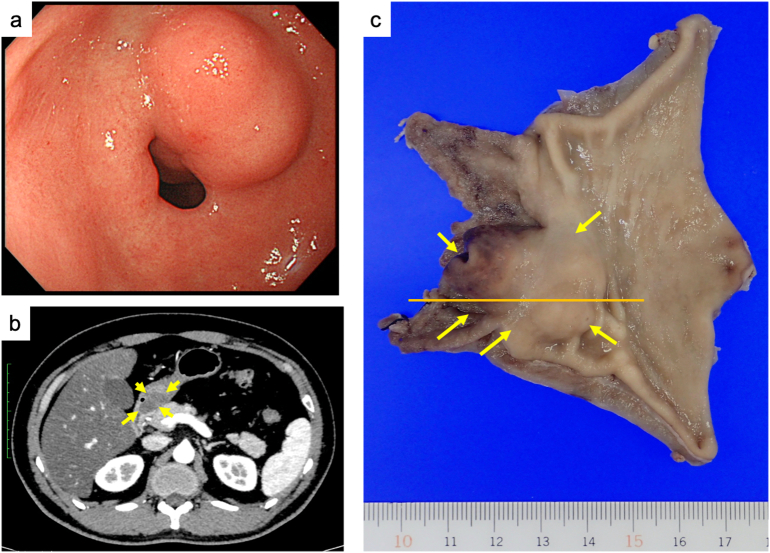
Gastrointestinal endoscopy revealing a submucosal tumor extending from the gastric pylorus through the duodenal bulb (a). Abdominal contrast-enhanced computed tomography showing a pyloric ring submucosal tumor measuring 33 × 19 mm (b). Gross pathology of the lesion (c). The orange line indicates the sectioned surface shown in Fig. 2. Yellow arrows indicate submucosal tumors in (a) and (c). (For interpretation of the references to colour in this figure legend, the reader is referred to the web version of this article.)

A 42-year-old man was referred to our institution for a pyloric ring submucosal tumor. The patient was asymptomatic and underwent upper gastrointestinal endoscopy as part of a regular check-up. A submucosal tumor lesion was incidentally detected on the posterior wall just before the pyloric ring of the stomach to the duodenal bulb (Fig. 1a). Contrast-enhanced computed tomography (CT) of the abdomen showed a 20 × 12-mm mass within the pyloric ring wall that was hypoabsorbable and homogeneous, likely benign (Fig. 1b). Approximately 10 months later, the tumor gradually increased in size. An explorative endoscopic biopsy was performed, but it did not lead to a definitive diagnosis because the tumor cells were not collected. Clinically, the preoperative diagnosis was a GIST or schwannoma. During the next 9 months of follow-up, the tumor grew to 33 × 19 mm, and laparoscopy-assisted pyloric gastrectomy with Billroth I anastomosis was performed (Fig. 1c). The tumor was completely removed pathologically at a distance of 2.9 cm for proximal margin length and that of 0.9 cm for distal margin length. The postoperative course was uneventful.

**Fig. 2:**
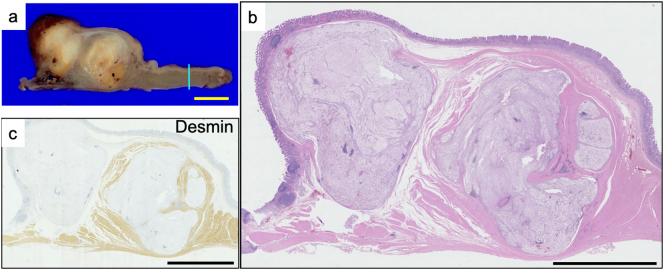
Macroscopic view of the tumor (a). Hematoxylin–eosin staining revealing a relatively well-demarcated, solid lesion (b) located in the lamina propria mucosae and submucosa (c). Scale bars: 1 cm.

**Fig. 3:**
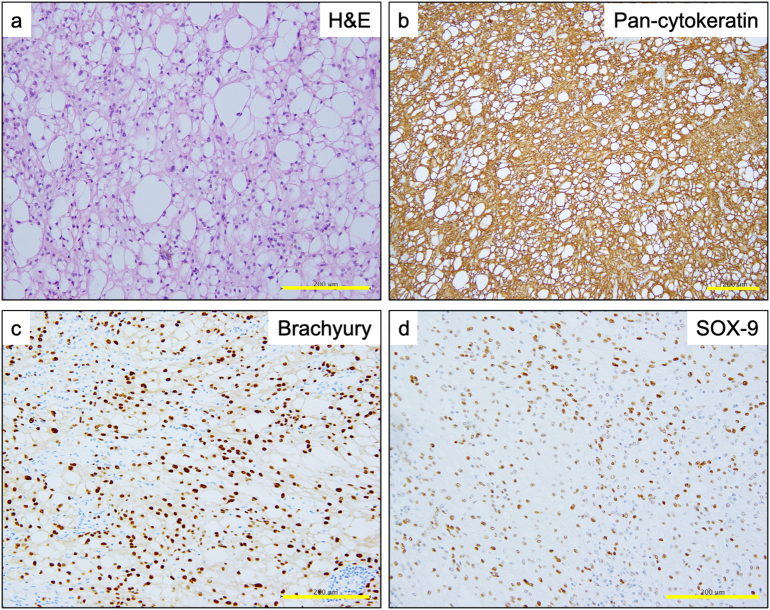
Photomicrographs of the tumor cells at a higher magnification. The tumor cells showing slightly mucinous vacuolated characteristics with mild nuclear atypia on hematoxylin–eosin staining (a). Diffuse strong cytoplasmic immunohistochemical staining for pan-cytokeratin (antibody clone [AE1/AE3]) and (b) diffuse strong nuclear immunohistochemical staining for brachyury (c) and SOX-9 (d).

Grossly, there was a relatively well-demarcated, solid lesion (Fig. 2a) located in the lamina propria mucosa and submucosa (Fig. 2b and c). Histological examination revealed proliferation of slightly mucinous vacuolated tumor cells with mild nuclear atypia (Fig. 3a). The tumor cells showed positive staining for the expression of pan-cytokeratin (AE1/AE3) (Fig. 3b), S-100, brachyury (Fig. 3c), and SOX9 (Fig. 3d) but negative staining for the expression of calretinin, D2-40, KIT, CD34, DOG-1, desmin, alpha-SMA, Melan-A, HMB45, and SOX10. Consequently, a pathological diagnosis of conventional chordoma was made [4]. No metastases, including perigastric lymph nodes, were identified histopathologically. Postoperative CT and magnetic resonance imaging (MRI) of the skull and all vertebrae every 6 months for a total of 18 months showed no neoplastic lesions, and the lesion was thus considered to be gastroduodenal in origin. No recurrence or metastasis was observed during the 1.5-year postoperative course. The patient did not undergo preoperative or postoperative chemotherapy or radiation therapy.

## 3 Discussion

Chordomas were first described by Virchow in 1857 as tumors with a histological appearance of pleomorphic cells with dark nuclei and vacuolated or granular cytoplasm [5]. The overall incidence of chordomas is estimated to be less than 10 per 10 million, with a higher incidence in men and individuals older than 40 years of age [6]. The primary site of 94% of chordomas is the spine, skull, or sacrum, while 6% have an extra-axial site [6]. Extra-axial–skeletal primary cases of the ulna, tibia, femur, and pubic bone have been reported [7], [8], [9]. Although extra-skeletal primary cases are exceptionally rare, they have been reported to occur in a wide variety of locations, including the toes, fingers, buttocks, chest wall, shoulders, thighs, hands, wrist joints, nasopharynx, and posterior mediastinum [7], [10], [11], [12], [13], [14]. No other reports on extra-skeletal chordomas originating in the gastrointestinal wall have been previously published in literature.

In the current case, microcystic/reticular schwannoma was the differential diagnosis based on hematoxylin–eosin (H&E) staining characteristics and S100 positivity [15]. However, the tumor was finally diagnosed as chordoma based on positive staining for the expression of brachyury, SOX9, and EMA and negative staining for the expression of SOX10. Brachyury, a nuclear T-box transcription factor, is a regulator of embryonic notochord development and has been proposed as the principal adjunct in chordoma diagnosis [7]. Although the morphological characteristics on H&E staining were almost identical to those of axial chordoma, the assumption that there is no primary chordoma in the intestinal wall made the diagnosis challenging. Staining for specific markers, such as brachyury and SOX9, is essential for an accurate diagnosis. Initially, the chordoma was considered a metastatic lesion from an occult primary tumor, but multiple postoperative MRIs of the area, including the skull and spine, revealed no neoplasms, leading to the conclusion that it was primary to the pyloric ring.

Chordomas are believed to arise from the vestigial notochordal tissue because they almost always arise in the axial skeleton and resemble the normal notochord [1]. The notochord origin hypothesis was originally discussed in terms of axial chordomas. Extra-skeletal soft tissue chordoma cases thus raise questions regarding histogenesis. There exists an argument as to whether ectopic notochordal tissue exists in soft tissue or particular molecular mechanisms alone can cause soft tissue chordoma without the presence of a notochord [1], [16], [17]. In the present case, no notochordal remnant or notochord-like tissue, including benign notochordal cell tumors, was histologically identified at the location of the tumor. No notochordal remnants have been reported in extraosseous soft tissue [10]. Soft tissue chordoma is extremely rare in all vertebrates, including humans [18]. Given this background, gastrointestinal chordoma, at least, in this case, may have developed through aberrant brachyury expression in the absence of notochord remnants.

Locoregional recurrence, rather than distant metastasis, is a common event following complete resection and represents a major clinical challenge [19]. More than half of patients experience recurrence after the initial surgical treatment [19]. Because a high proportion of recurrence events occur even 10 years after complete surgical resection, long-term follow-up with MRI of the primary tumor site for the first 3–5 years after diagnosis is recommended [19], [20]. To prevent recurrence, it is important to perform complete resection with sufficient margins to ensure no residual tumor. In this case, the follow-up period was only 1.5 years; thus, the possibility of recurrence could not be excluded. However, compared with chordomas arising in the bone or connective tissue, intestinal chordomas are relatively easy to resect with sufficient margins as long as they do not reach the serous surface, which may be advantageous in preventing recurrence.

Some limitations of the current report should be mentioned. First, given that a long-term follow-up period of around 5 years is recommended, the 1.5-year follow-up period, in this case, is relatively short to conclude that there is no recurrence. Second, we did not perform comprehensive genomic profiling to detect somatic variants or chromosomal rearrangements specific in the tumor cells. Extended molecular profiling may yield insight into how gastrointestinal chordomas differ from common axial–skeletal chordomas in terms of drug targeting and disease entity classification.

## 4 Conclusion

In this report, we describe the first documented case of chordoma arising within the gastrointestinal wall, which is a novel site of development for this rare neoplasm. The tumor was completely removed surgically without postoperative recurrence within the 1.5-year follow-up period. No chemotherapy or radiation therapy was administered. Analysis of H&E characteristics and positive staining properties, including those of brachyury and SOX9, is essential for an accurate diagnosis. Although it is difficult to determine whether the tumor, in this case, arised from the notochord, no notochordal remnants were identified in the location of the tumor. Owing to their easy resection with sufficient margins, gastrointestinal chordomas may have the advantage of avoiding recurrence.

## Provenance and peer review

Not commissioned, externally peer-reviewed.

## Consent

Written informed consent was obtained from the patient for publication of this case report and accompanying images. A copy of the written consent is available for review by the Editor-in-Chief of this journal on request.

## Sources of funding

This research did not receive any specific grant from funding agencies in the public, commercial, or not-for-profit sectors.

## Ethical approval

This study was exempted from ethical approval.

## Author contribution

Ryotaro Hashizume: Board-certified pathologist who made the diagnosis, and was involved in the conceptualization, methodology, investigation, formal analysis, and writing of the original draft.

Shinsuke Matsuda: Board-certified surgeon performed the operation, methodology, investigation, and formal analysis.

Moritaka Nagai: Involved in the methodology and investigation.

Kazuki Hirata: Clinical laboratory technician who prepared tissue specimens and helped with the investigation.

Hiroshi Imai: Board-certified pathologist who made the diagnosis, conceptualization, methodology, investigation, and formal analysis.

Ryoji Kushima: Board-certified pathologist who made the diagnosis, methodology, investigation, formal analysis, and reviewed and edited the manuscript.

## Research registration

This study was not a first in man study.

## Guarantor

Ryotaro Hashizume, MD, PhD.

Ryotaro Hashizume accepts full responsibility for the work and/or the conduct of the study article, providing full access to data, and controlling the decisions about the publishing of the paper.

## Declaration of competing interest

The authors have no conflicts of interest to disclose.
